# Duplicate Publications: Time to Ring Alarm Bells

**DOI:** 10.4103/0970-0218.62558

**Published:** 2010-01

**Authors:** Anurag Chaudhary, Sarit Sharma, Sangeeta Girdhar, Mahesh Satija

**Affiliations:** Department of Community Medicine, Dayanand Medical College and Hospital, Ludhiana, Punjab, India. E-mail: anuragdmc@yahoo.co.in

Sir,

A redundant or duplicate publication is a publication of a scientific paper that overlaps substantially with one already published.(1)

As per “Guidelines on Good Publication Practice,”(2) the Committee on Publication Ethics (COPE) defined that a redundant publication occurs when two or more papers, without full cross references, share the same hypothesis, data, discussion points or conclusions.

According to Reeves *et al*., the defining characteristics of a duplicate publication is that in addition to the above, it shares at least some of the same authors.(3)

As an example, the article titled “Fertility profile and its correlates in a rural population of Dehradun district” was published as letter to editor in *Indian Journal of Community Medicine* (IJCM 2007; 32(2):152-3) and as original research article in *Indian Journal of Preventive and Social Medicine* (Ind J PSM 2007;38(3):142-49).

An act of Professional Misconduct-why?

The reader after reading the same article in two different journals may falsely substantiate the same observation and place greater reliance on evidence that appears to have been confirmed in more than one study. Moreover, it leads to wastage of valuable space in the journal as well as precious time of the editor. As a consequence, a deserving article might get rejected.

Also, the results of meta-analysis might get biased because of the same observation being repeated in two different journals. Tramer *et al*. concluded in their study that inclusion of duplicated data in meta-analysis led to a 23% overestimation of Ondansetron's antiemetic efficacy.(4)

However, some persons are of the view that as journals have different readership, this leads to wider accessibility of the scientific materials to the readers,(5) but in this era of electronic/internet revolution this point seems to be invalid.

Possible reason: The most important reason may be deliberate or intentional duplication of the research article in order to increase the number of publications that is unfortunately a basic requirement for career progression in medical profession. Similar apprehensions are also expressed by Dr. AJ Singh in his article titled Plagiarising Plagiarism.(6)

Recommended actions: In the light of above discussion, it is evident that the problem of duplicate publications is an issue that needs immediate attention on the part of both editors and readers. The actions to be taken in such cases range from retracting the article to issuing a ban on further publications of such authors.

According to COPE Guidelines, the editor has to investigate and label the misconduct as serious or less serious followed by decision on what action to be taken when such misconduct is noticed or reported. The Guidelines provide a list of Sanctions that may be applied separately or combined ranging from a letter of warning to as serious as reporting the case to the General Medical Council or other such authority for appropriate action.

Safeguards

- Increasing awareness among authors and readers regarding this issue.
- Before submitting an article for publication, the authors should consult the ‘Uniform Requirements for Manuscripts submitted to Biomedical journals.(7)
- Prompt reporting of such misconduct is the moral responsibility of the readers as well.
- Most importantly, the editors have a pivotal role to play in prevention of duplicate publications.
